# 1,2,3-Triazolium macrocycles in supramolecular chemistry

**DOI:** 10.3762/bjoc.15.211

**Published:** 2019-09-12

**Authors:** Mastaneh Safarnejad Shad, Pulikkal Veettil Santhini, Wim Dehaen

**Affiliations:** 1 Molecular Design and Synthesis, Department of Chemistry, KU Leuven, Celestijnenlaan 200F, B-3001 Leuven, Belgium

**Keywords:** anion recognition, catenane, chalcogen bonding, click reaction, molecular reactor, hydrogen bonding, pH sensor, rotaxane, supramolecular, 1,2,3-triazolium macrocycles

## Abstract

In this short review, we describe different pathways for synthesizing 1,2,3-triazolium macrocycles and focus on their application in different areas of supramolecular chemistry. The synthesis is mostly relying on the well-known “click reaction” (CuAAC) leading to 1,4-disubstituted 1,2,3-triazoles that then can be quaternized. Applications of triazolium macrocycles thus prepared include receptors for molecular recognition of anionic species, pH sensors, mechanically interlocked molecules, molecular machines, and molecular reactors.

## Review

### 1. Introduction

Supramolecular chemistry – “The chemistry beyond the molecule” [1] – is an ever growing interdisciplinary area has emerged from the early host–guest chemistry to more elaborate bio-inspired supramolecular aggregates by exploiting various noncovalent interactions such as hydrogen bonding, π–π stacking, electrostatic interactions, van der Waals forces, hydrophobic/solvatophobic effects and coordination bonds [2–3]. Advances in supramolecules from molecular to macroscopic size with pre-structured or functionalized receptors and multivalent binding positions have led to applications in molecular recognition, sensing, molecular machines and devices, supramolecular polymers, stimuli-responsive materials, supramolecular catalysis and drug-delivery systems [4–7].

Inspired by the attractive role of various N-heterocyclic building blocks such as imidazoles [8–9], polypyrroles [10–11], and indole moieties [12–13], as part of supramolecular receptors, triazole heterocycles containing macrocycles have recently been introduced as new host molecules for the selective recognition of ions, mechanically interlocked molecules (MIMs), supramolecular polymers etc. [14–15]. Noncovalent interactions play a dynamic role in the binding mechanism of triazoles as macrocyclic receptors. It has been reported that the combined effects of both an electron lone pair on the nitrogen of the heterocycle and the acidic C5–H proton make 1,2,3-triazoles interesting candidates for amide bond surrogates. Interestingly, 1,2,3-triazole units can act as sensors for both anions and cations via different binding mechanisms [16–17]. The heterocyclic ring N2 and N3 atoms could realize the selective recognition of the cations whereas the C5–H···anions electrostatic interaction results in the sensing of anions. In fact, strictly speaking this interaction is not a hydrogen bonding interaction seen as there is no X···H–Y unit (X, Y = O, N, F) but these interactions are often referred to as such in the literature.

**Figure 1:**
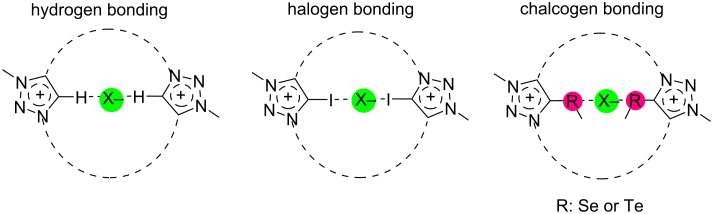
Hydrogen, halogen or chalcogen bonding to anions within a bistriazolium macrocycle.

The anion binding can be enhanced by the alkylation of 1,2,3-triazole units to more electrophilic 1,2,3-triazolium units by influencing both hydrogen bonding-like and anion–π interactions. Moreover, halogen bond (XB) and chalcogen bond (ChB) interactions (see Figure 1) also been applied for the selective detection of anions by exchanging C5–H protons with halogens (iodine, bromine) and chalcogens (selenium and tellurium) [18].

**Figure 2:**
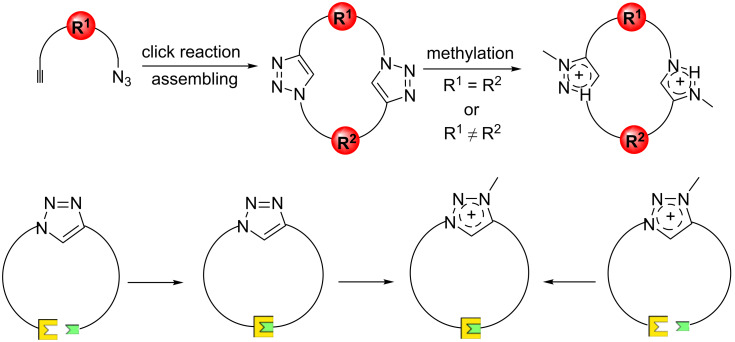
Main synthetic strategies towards macrocyclic triazoliums.

While there are several strategies for the synthesis of triazoles, the Cu(II)-catalyzed azide–alkyne cycloaddition reaction (CuAAC click reaction) is considered as one of the most efficient, simple and mild approaches towards the preparation of 1,4-disubstituted 1,2,3- triazole units [19–24]. Macrocyclic ring closure can be achieved by the CuAAC of building blocks functionalized with both azide and alkyne, using [1 + 1], [2 + 2], [*n* + *n*] strategies depending on how much triazoles are needed to be included in the macroring. A second strategy is the macrocyclization of an acyclic precursor containing pre-functionalized triazole by other ring closure methods such as Grubbs metathesis [25] and amidation reactions. The macrocyclization is normally followed by N-alkylation (often methylation) leading to the synthesis of highly functionalized 1,2,3-triazolium macrocycles under optimized reaction conditions, although cyclizations also have been carried out starting from triazolium building blocks (Figure 2). Using a small variation in the reaction protocols of the click reaction, iodine atoms can be introduced at the 5-position of the triazoles (triazolium), and nucleophilic substitution of halogen can be used to introduce chalcogens (Se, Te). Thus, the corresponding triazolium macrocycles can take advantage respectively of halogen and chalcogen bonding as part of the molecular recognition (vide infra).

In this review, we highlight the recent advances in the synthesis and applications of triazolium macrocycles as anion sensors, molecular reactors, and pH sensors through the description of selected examples. The macrocycles involved vary from having relatively simple structures to being part of different types of mechanically interlocked molecules and molecular machines. We will not concern ourselves in this review with the applications of metal complexes based on N-heterocyclic carbene coordination chemistry, derived of triazoliums or with applications as ionic liquids [26–28].

### 2. Anion recognition

Due to the critical role of negatively charged species in numerous biological, environmental, chemical and medicinal processes, anion receptors and transporters play an important role in supramolecular chemistry [17,29].

Thus, diverse anion receptors based on 1,2,3-triazolium recognition elements have been reported in the literature in which they bind to the anionic species by utilizing multiple noncovalent interactions based on electrostatics, including hydrogen bonding (HB), anion–π interactions [30], and on Lewis acidity/basicity [31].

#### 2.1. Bile acid-based 1,2,3-triazolium macrocycles

Bile acid-based macrocycles are a class of steroid-based receptors which can host polar molecules in nonpolar solvents because of their amphiphilic nature. As a result, their design and synthesis has been a topic of substantial research interest in supramolecular chemistry [32–33].

Various synthetic methods have been developed for the synthesis of bile acid-based macrocycles, using macrolactamization, ring-closing metathesis and Ugi-multicomponent macrocyclization. Developing more efficacious and economical methods for the construction of these macrocycles is still in demand due to the disadvantages of the aforementioned methods such as low yield of cyclization, long synthesis, and sometimes, usage of costly precursors [34–35].

**Figure 3:**
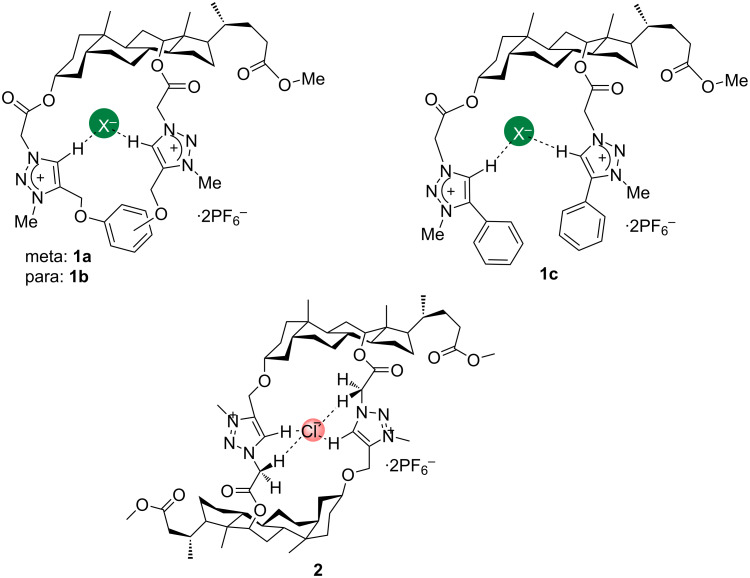
Chemical structure of compound **1** (**1a**, **1b** and **1c**) and **2**.

Pandey et al. have synthesized two new bile acid-based macrocycles containing two 1,2,3-triazolium moieties (**1a** and **1b**) and studied their anion bonding properties by ^1^H NMR spectral changes in the CDCl_3_ solution of anionic species (Figure 3). The receptor **1a** has shown remarkable affinity toward fluoride ion with an association constant of 560 M^−1^ followed by Cl^−^ > Br^−^ > I^−^ > CH_3_COO^−^ with the affinity range of 5.6 × 10^2^ to 6.0 × 10^1^ M^−1^ but no binding was observed with the H_2_PO_4_^−^ ion. On the other hand, the observed affinity trend of receptor **1b** towards anions is H_2_PO_4_^−^ (1.1 × 10^3^ M^−1^) > Cl^−^ (6.90 × 10^2^ M^−1^) > Br^−^ (4.5 × 10^2^ M^−1^) > F^−^ (3.7 × 10^2^ M^−1^) > I^−^ (2.0 × 10^2^ M^−1^) > CH_3_CO_2_^−^ (2.5 × 10^1^ M^−1^) in which the affinity of the receptor **1b** towards H_2_PO_4_^−^ could be attributed to the larger size of the receptor cavity due to the *para*-substituted benzene ring. Interestingly, the corresponding acyclic receptor **1c** has showed a higher affinity toward the H_2_PO_4_^−^ ion (1.9 × 10^3^ M^−1^) compared to the cyclic ones because of the high flexibility of the acyclic receptor for accommodating the bulky anion. The observed selectivity trend of **1c** was H_2_PO_4_^−^ > Cl^−^ > F^−^ > Br^−^ > I^−^ > CH_3_COO^−^ with the dissociation constants 1.9 × 10^3^, 3.9 × 10^2^, 3.6 × 10^2^, 2.0 × 10^2^, 1.0 × 10^2^ and 3.0 × 10^1^ M^−1^, respectively [36].

Conversely, another bile acid-based macrocycle **2** which was synthesized by the same group has demonstrated a very high selectivity toward the chloride anion (*K*_a_ = 3.7 × 10^3^ M^−1^) followed by HSO_4_^−^ > H_2_PO_4_^−^ > F^−^ > Br^−^ > CH_3_COO^−^ > I^−^ with the dissociation constants of 8.0 × 10^2^, 6.5 × 10^2^, 4.0 × 10^2^, 9.0 × 10^1^, 6.0 × 10^1^ and 5.0 × 10^1^ M^−1^, respectively, in CDCl_3_ at 298 K. The significant selectivity of this receptor for chloride anion is due to the cavity size (see Figure 3) [37].

#### 2.2. Optical anion sensing by 1,2,3-triazolium macrocycles within porphyrin cages

Various porphyrin-based host supramolecules containing hydrogen-bond donor groups such as integrated amide, urea, pyrrole, ammonium and imidazolium which can recognize different anions, have been reported in the literature. As a matter of fact, porphyrin macrocycles are used for sensing of anionic compounds by a measurable physical response due to their intrinsic optical and redox properties [38].

**Figure 4:**
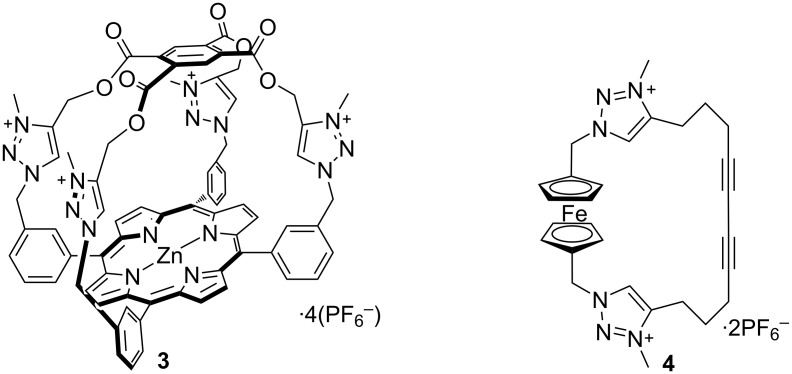
Chemical structure of compound **3** and **4**.

Beer and co-workers have synthesized the novel tetra-1,2,3-triazolium zinc porphyrin cage **3** (Figure 4) and have probed its characteristics by using UV–visible spectroscopy, determining the association constants for complex formation in 5% water/acetone. This receptor has shown affinity toward all of the halide ions and especially oxoanions with the strongest binding for sulfate dianion [39]. The observed affinity trend for this receptor was SO_4_^2−^ (5.2 × 10^5^ M^−1^) > AcO^−^ (1.3 × 10^5^ M^−1^) > H_2_PO_4_^−^ (1.2 × 10^5^ M^−1^) > F^−^ (8.7 × 10^3^ M^−1^) > Cl^−^ (7.4 × 10^3^ M^−1^) > Br^−^ (<5.0 × 10^1^ M^−1^) ≈ I^−^ (<5.0 × 10^1^ M^−1^). These authors have also studied the association constant of macrocycle **3** in the more competitive 15% water/acetone medium, still demonstrating a high binding affinity for sulfate anion (*K*_a_ = 2.5 × 10^5^ M^−1^) while the cage **3** did not show any binding toward the other tested anions in the more aqueous solvent mixture.

#### 2.3. Redox-active 1,2,3-triazolium receptors

A ferrocene-containing dicationic bis-triazolium macrocycle **4** (see Figure 4) has been designed and synthesized by utilising the intramolecular Eglinton cyclisation of an acyclic bis(triazolylalkyne)ferrocene precursor followed by alkylation. The anion sensing ability was investigated by ^1^H NMR titration experiments in CD_3_CN solution and cyclic voltammetry in 0.1 M TBA·PF_6_ (TBA = tetrabutylammonium) in CH_3_CN (Figure 3). WinEQNMR2 analysis of the titration data has revealed that among the tested anions, the receptor **4** binds strongly with chloride and benzoate ions through favorable charge-assisted C–H···anion interactions. According to the cyclic voltammetry analysis, the redox-active macrocycle was able to recognize chloride, causing a cathodic shift of the *E*_pa_ wave of the ferrocene/ferrocenium redox couple. However, a quick disappearance of the redox signal was observed when the analogous experiment was done with benzoate, due to the precipitation of an insoluble ferrocenium complex [40].

#### 2.4. Mechanically interlocked catenanes containing 1,2,3-triazolium macrocycles

Mechanically interlocked molecules like rotaxane and catenane have attracted much attention in the field of nanoscale molecular machines and switches [41] and their importance has been recognized by the 2016 Nobel prize. Anion recognition can be used as an impediment for rotary movement in the rotaxane structure. Besides, catenanes possessing a mechanically interlocked ring, have attracted considerable attention as components of functional molecular devices, such as switches [42–43], unidirectional motors [44], electronic displays [45] as well as in anion recognition [46].

**Figure 5:**
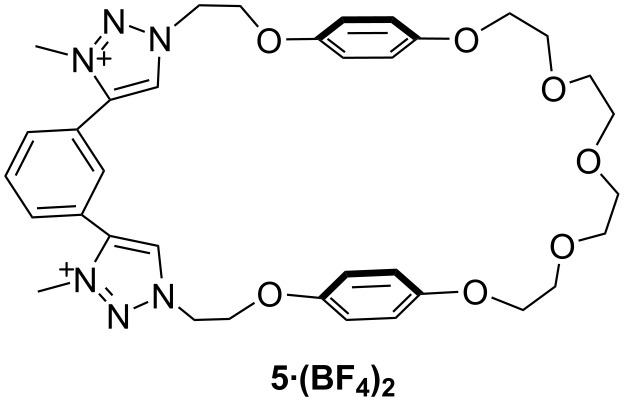
Chemical structure of compound **5**.

Interestingly, Beer et al. have attempted the synthesis of tetratriazolium hetero-catenane, but the purification and isolation was felt to be very difficult to further proceed. However, the anion binding property of the corresponding bistriazolium macrocyclic part **5·(BF_4_)_2_** (Figure 5) has successfully been investigated using ^1^H NMR titration experiments in CD_3_CN. The highest binding affinity was found to be almost similar for both BzO^−^ and Cl^−^ (4.7 × 10^2^ and 4.6 × 10^2^ M^−1^, respectively) then followed by Br^−^ (2.1 × 10^2^ M^−1^), N_3_^−^ (1.9 × 10^2^ M^−1^) and I^−^ (1.3 × 10^1^ M^−1^) [47].

**Figure 6:**
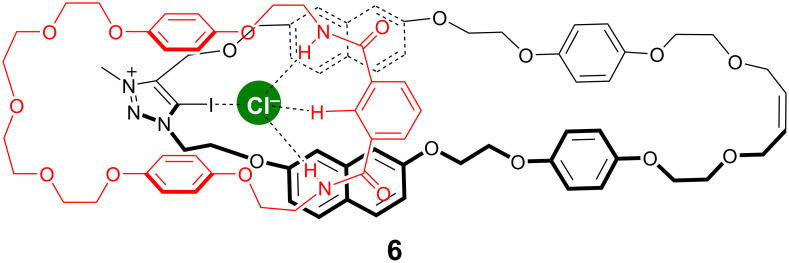
Chemical structure of compound **6**.

Soon after, a mixed halogen and hydrogen bonding hetero[2]catenane **6** (Figure 6) was successfully synthesized by the same group via an anion templated Grubbs’ II-catalyzed RCM clipping mechanical bond forming methodology [48]. The ^1^H NMR spectroscopy in CDCl_3_ (293 K, 500 MHz) and the fluorescence titration experiments which were done in acetonitrile have demonstrated that the synthesized 1,2,3-triazolium macrocycle **6** was able to bind and sense several anions but the highest affinity of this receptor was for acetate (*K*_a_ = 1.5 × 10^5^ M^−1^) and dihydrogen phosphate (*K*_a_ = 4.5 × 10^4^ M^−1^).

**Figure 7:**
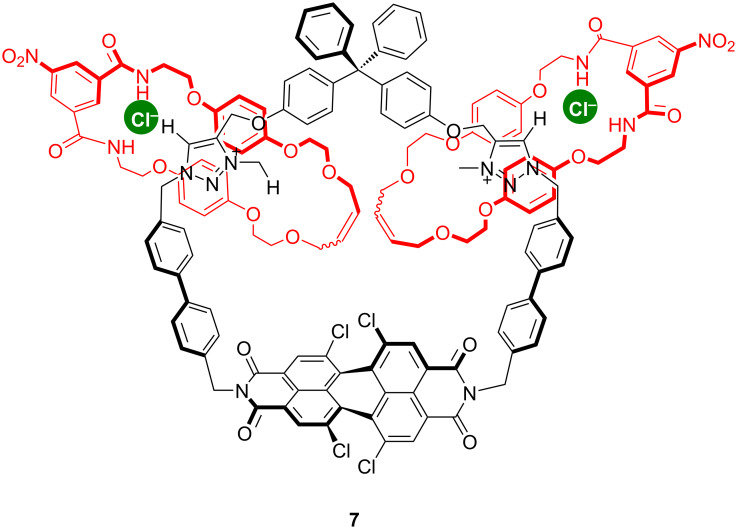
Chemical structure of compound **7**.

Because of the excellent properties of perylene diimide (PDI) as a chromophore, fluorophore, or redox center, this unit has become increasingly popular in the construction of supramolecular materials. Interestingly, a dynamic [3]catenane **7** (Figure 7) consisting of a large four station central PDI macrocycle with two triazolium motifs and two smaller isophthalimide containing macrocycle has been designed and synthesized by Beer’s group [49]. An anion induced circumrotatory motion of the smaller rings from the core tetrachloro substituted PDI motifs containing triazolium groups has been utilized for the selective colorimetric and fluorescence anion sensing in competitive protic organic and aqueous-organic solvent media. By addition of tetrabutylammonium (TBA) chloride to a solution of **7** in CHCl_3_/CH_3_OH (3:1, v/v), a remarkable naked-eye recognizable color change from red to orange was observed. According to fluorescence spectroscopy, the emission intensity of PDI increased considerably (quantum yield enhancement factor of 57%). Anions are recognizable by catenane **7** at low concentration (10^−5^ mol/L) in a competitive aqueous–organic CHCl_3_/CH_3_OH/H_2_O mixture (45:45:10, v/v/v). Fluorescence spectroscopy measurements supported by molecular dynamics simulation data have revealed that the smaller macrocyclic rings move from the core-substituted PDI motif to the two 1,2,3-triazolium groups due to the anion binding, including chloride and other oxoanion salts. They also proved that the anions binding strength falls in line with their basicity as seen in the series AcO^−^ > H_2_PO_4_^−^ > Cl^−^ > SO_4_^2−^ >NO_3_^−^.

#### 2.5. 1,2,3-Triazolium macrocycles and [2]rotaxanes

Supramolecular interactions could happen between Lewis bases and electron-defective heavy chalcogen atoms containing Lewis acidic s-holes which is known under the term chalcogen bonding (ChB) [18].

**Figure 8:**
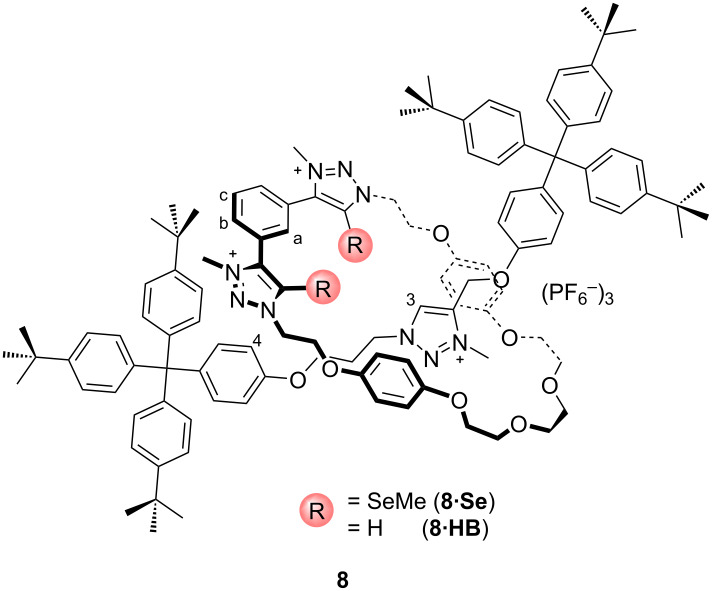
Chemical structure of compound **8**.

A mechanically interlocked rotaxane **8** (Figure 8) has been prepared by Beer and co-workers utilizing the 5-(methylchalcogeno)-1,2,3-triazole (chalcogen = Se, Te) motif as a novel ChB donor for anion binding. By exploiting the possibility of the chalcogen atoms to orient within the macrocycle cavity to chelate the copper(I) endotopically, the first examples of mechanically interlocked [2]rotaxanes containing ChB donor groups has been prepared via an active metal template strategy. The ^1^H NMR binding data of **8·Se** were studied in organic and aqueous solvent mixtures, revealing that ChB–anion binding affinity can compete with and even outperform hydrogen bonding receptor analogues. Due to the larger degree of covalency with the heavier halides, charge-assisted ChB-mediated anion binding prefers larger halides as in the series I^−^ > Br^−^ > Cl^−^. However, this chalcogen bonding 1,2,3-triazolium macrocycle was not able to host larger anions such as oxoanions due to the smaller size of the cavity [50]. In addition, they have also shown considerable differences in anion recognition behavior of **8·Se** in comparison with chalcogen-free host analogues **8·HB** demonstrating that the **8·HB** rotaxane can host all of the tested anions within the interlocked cavity. Regarding this, it can be concluded that the bulky methylselenium subunits of rotaxane **8·Se** creates steric restriction for bigger anionic species such as sulfate and acetate and leads their binding to the outside of the cavity.

#### 2.6. Multi-1,2,3-triazolium macrocycles

As a matter of fact, a cyclic host framework incorporating multiple charged moieties may create a potent anion receptor that functions in aqueous/organic solvent mixtures [51].

**Figure 9:**
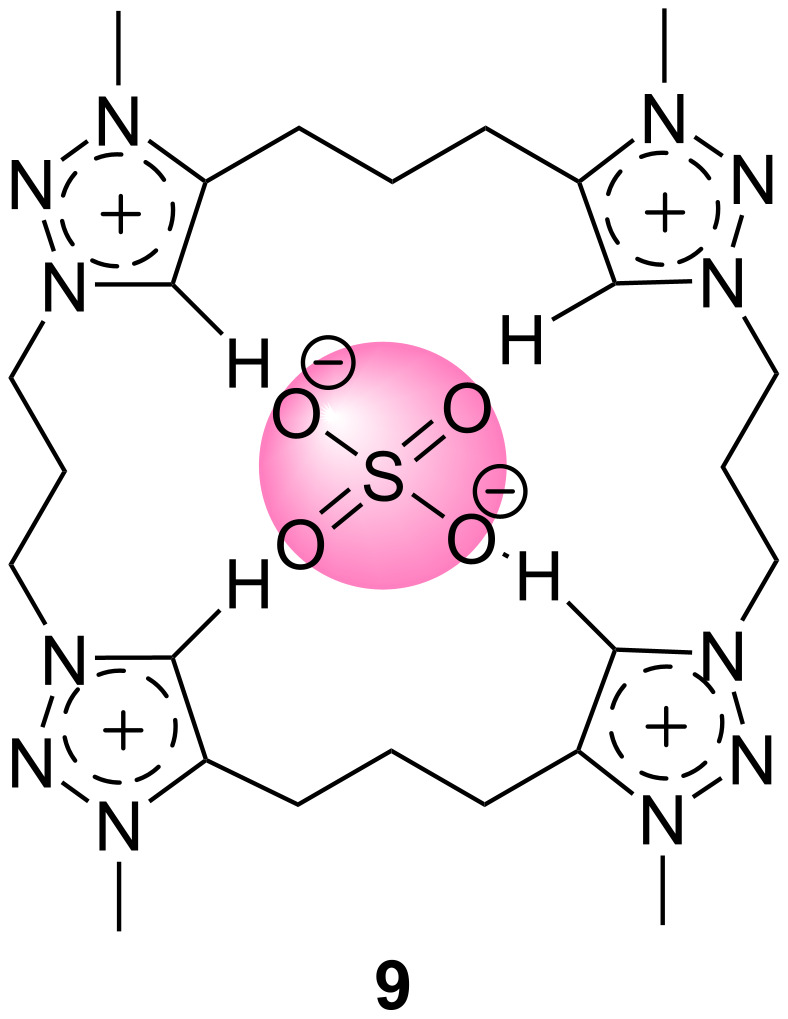
Chemical structures of compound **9**.

A tetra-1,2,3-triazolium macrocycle **9** (Figure 9) was reported in 2012 by the Beer group. The synthesis was realized by using a CuAAC cyclisation of triazole bisazides and bisalkynes and subsequent alkylation. The charge-assisted C–H···anion interaction is used by this receptor for binding strongly to the anionic species. Among all of the tested negatively-charged ions such as halides and oxoanions, receptor **9** recognizes iodide and bromide stronger than chloride, fluoride, acetate and sulfate in a 9:1 DMSO-*d*_6_/D_2_O solvent mixture. The highest displayed binding affinity by receptor **9** was towards sulfate dianion (*K*_a_ = >10^4^ M^−1^) out of all the tested anions found the trend was I^−^ > Br^−^ > Cl^−^ > F^−^ > OAc^−^ with the dissociation constants of 4.6 × 10^2^, 3.9 × 10^2^, 2.3 × 10^2^, 2.3 × 10^2^ and 1.5 × 10^2^ M^−1^, respectively. By carrying out extensive molecular modelling analysis which gives information about solution anion binding trends, the authors found that the cavity size of macrocycle, solvent effects and strength of electrostatic interactions notably affect the observed anion recognition processes [52].

**Figure 10:**
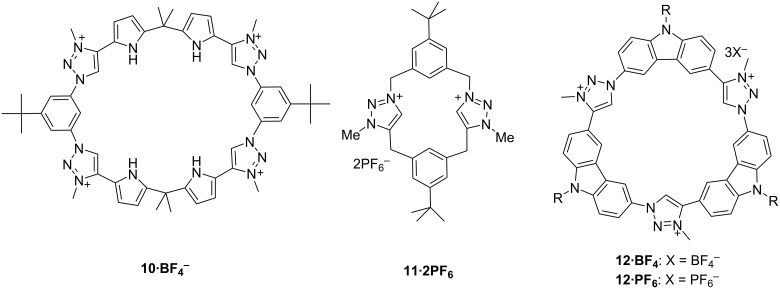
Chemical structures of compound **10**, **11** and **12**.

**2.6.1. 1,2,3-Triazolium macrocycles with additional H-bond donors:** The benefits of combining the triazolium motif with other hydrogen bond donor motifs has been explored by Sessler et al. and they have synthesized a pyrrole based tetra-1,2,3-triazolium macrocycle **10** (Figure 10) via the tetraalkylation of a triazole macrocycle originally prepared via click chemistry. Different hydrogen bond donors such as pyrrole N–H and benzene C–H along with the triazolium C5–H have been successfully incorporated within a single macrocyclic framework. The complex formation of anions with the hydrogen bond donors varied according to the polarity of the solvent. It was found that the binding constants for interaction with monoanions become smaller as the polarity of the solvent medium increases on moving from acetonitrile to methanol. Among all of the tested anions, macrocycle **10** has shown a high selectivity for pyrophosphate HP_2_O_7_^3−^, and tetrahedral oxyanions HSO_4_^−^, H_2_PO_4_^−^ relative to various tested monoanions and trigonally planar anions in mixed polar organic–aqueous solvent media. Both experimental and theoretical results support the counterintuitive conclusion that triazolium (CH)^+^–anion interactions are less important in an energetic sense than neutral aromatic CH–anion interactions in polar solvents. An important implication for receptor design has been revealed by combining various hydrogen bond donor motifs, particularly systems designed to recognize anions in highly polar organic media or aqueous environments by considering of different solvent dependence of the fundamental hydrogen bonding and electrostatic interactions related to the individual recognition motifs [53].

Another cyclophane macrocycle containing two 1,2,3-triazolium moieties was synthesized by Alcalde and co-workers in a moderate yield [54]. The anion-binding behavior of receptor **11** (Figure 10) was examined with chloride, acetate, cyanide and dihydrogenphosphate anions by ^1^H NMR titrations in CD_3_CN, DMSO-*d*_6_ or CDCl_3_ using the corresponding salts. The titrations of triazolium macrocycle **11** with acetate showed that the bis(imidazolium) receptor **11** underwent a de-shielding of its C(5)–H heteroaromatic proton (Δδ H(c) ≈ 2 ppm) in CDCl_3_ or DMSO-*d*_6_ solvent. The Job’s plot analysis of titration of **11**, in CD_3_CN, revealed a 1:1 receptor to anion binding stoichiometry. It has also been proven that the presence of the 1,2,3-triazolium unit decreases the anion recognition ability in CD_3_CN in comparison with its bis(imidazolium) analogue. Given that, the highest affinity of this heterophane system was toward acetate (*K*_a_ = 5.0 × 10^3^ M^−1^) in CD_3_CN.

Although the iodide ion is biologically important because of its indispensable role in thyroid gland function, among all of the reported receptors in the literature there are still challenges to find selective iodide receptors due to its low basicity, large size and low charge density. There is an expectation that the combination of strong hydrogen-bonding sites and a large cavity could cause strong complexation with larger halide ions [55]. In 2016 a macrocycle has been reported by Nakamura and his co-workers containing three 1,2,3-triazolium and carbazole moieties **12** (Figure 10). π–π stacking interactions and the self-association ability of the synthesized macrocycle are increased in comparison with its neutral analogue due to the decreased electron density of the triazolium derivative. Rigid macrocycles **12** display selective complexation with I^−^ because of the complementary size of the cavity of the macrocyclic ring and the iodide ion. In addition, the acidity of the inner protons of **12** is higher than the corresponding neutral analogue which also helped to increase the ability of complexation of **12** with iodide [56].

#### 2.7. Functional molecular crystal and materials

**Figure 11:**
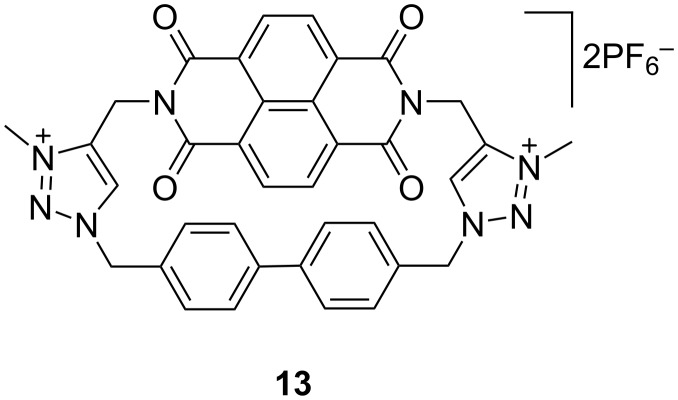
Chemical structure of compound **13**.

Combining anion–arene interactions and controlling the electron-transfer or charge-transfer process concerning an anionic guest by using a cyclophane is uncommon [57] but can be realized by inserting a photoactive binding motif into a cyclophane [58]. In this regard, Li et al. have synthesized a cationic cyclophane **13** (Figure 11) based on an electron poor naphthalenediimide (NDI) moiety and cationic 1,2,3-triazolium units. Cyclophane **13** was employed to control the interactions between anions and the NDI motif [59]. NDI was chosen due to its excellent photochemical and electrochemical properties. In addition, the electron deficiency and aromatic nature of NDI is important for anion–π interactions [60] to control the charge-transfer properties.

It also has been observed that in the solid state the movement of the molecules is restricted because of charge-transfer complexes between NDI and Cl^−^, Br^−^ or I^−^. Because macrocycle **13** precipitated in less polar organic solvents upon addition of halides, the binding affinities were studied in DMSO in which the binding constants for Cl^−^, Br^−^ and I^−^ were found as 4.4 × 10^1^ M^−1^, 1.7 × 10^1^ M^−1^, 8.5 M^−1^, respectively. Thus, the interaction of 1,2,3-triazolium CH is decreasing with increasing halide size. Besides, the complexes of halides with the macrocycles were shown to have visible charge-transfer absorptions in the molecular crystals. Thus, these complexes could be useful for designing functional molecular crystals and materials which can be applied for the study of photoinduced electron transfer and energy conversion towards application in the field of molecular electronics.

### 3. Molecular reactors

Designing synthetic host systems capable of binding to specifically targeted substrates by imitating various chemical processes is one of the most substantial objectives in chemistry. Encapsulation of two or more substrates within the inner space of the host speeds up and controls the reaction because of a significant increase of the effective molarity and the favorable orientation of the substrates inside the cavity [61].

**Figure 12:**
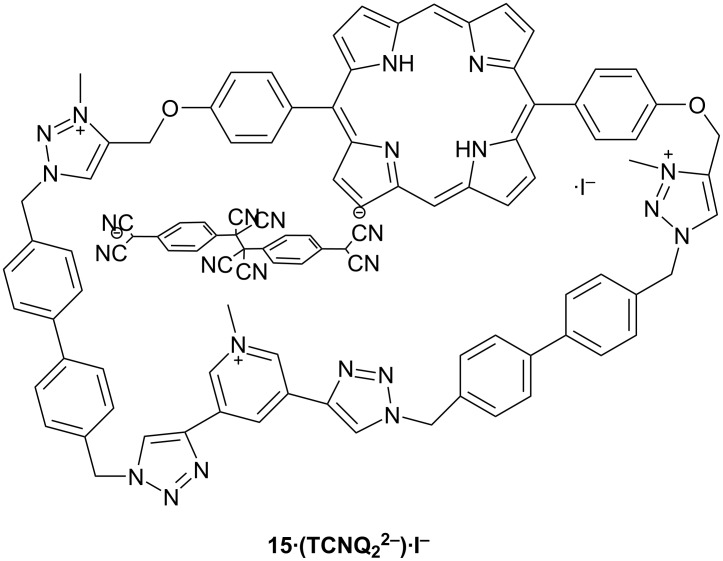
Chemical structure of compound **15** including the sigma-connected TCNQ dimer.

Thus, a nanometer-sized macrocyclic receptor based on a photoactive porphyrin unit and anion-binding pyridinium and 1,2,3-triazolium units **15** was reported by Li and co-workers This receptor was synthesized via well-known “click chemistry”, followed by triple quaternization of two triazoles and one pyridine unit (Figure 12) [62]. This macrocycle (in its hexafluorophosphate form) binds three equivalents of acetate anions in acetonitrile solvent, according to the Job plot based on UV spectra at 365 nm.

More interestingly, based on the results and control studies, Li’s group have proposed the ability of macrocycle **15** (triiodide form) to act as a nanoreactor by the photo-driven dimerization of tetracyano-*para*-quinodimethane (TCNQ). The proposed mechanism consists of 7 steps:

1. the formation of a 1:1 host–guest [15·TCNQ]^2+^ complex by pre-reduction to a [TCNQ]^−·^ anion radical in situ, and encapsulation in the host,
2. photochemical excitation of the porphyrin unit in the reactor,
3. photoinduced electron transfer from the excited porphyrin unit to a neutral TCNQ, giving rise to a porphyrin radical cation and a second [TCNQ]^−·^ radical anion,
4. dragging the second [TCNQ]^−^ near to the cavity by the help of the electrostatic stabilization supplied by the 1,2,3-triazolium cations,
5. coupling of the two [TCNQ]^−·^ anion radicals in the cavity and rapid formation of the sigma-connected dimer,
6. reduction of the photogenerated porphyrin cation by the iodide counterions of the triazolium macrocycle, either before or after the constraining of the second [TCNQ]^−·^ anion radical,
7. finally, the dimer is either ejected from the host or is displaced by the [TCNQ]^−·^ anion radical.

It was clearly shown that the macrocycle **15** acts as a nanoreactor because there is an acceleration of the dimerization by two orders of magnitude and the turnover of the catalyst was demonstrated.

The synthesis of cyclic carbonates from epoxides and CO_2_ is an efficient method for CO_2_ fixation. The development of an effective chiral catalyst for the efficient kinetic resolution of epoxides with CO_2_ remains a big challenge. Recently, Ema and co-workers described a chiral binaphthyl strapped Zn(II) porphyrin with triazolium halide units as a bifunctional catalyst for the kinetic resolution of epoxides with CO_2_ [63].

**Figure 13:**
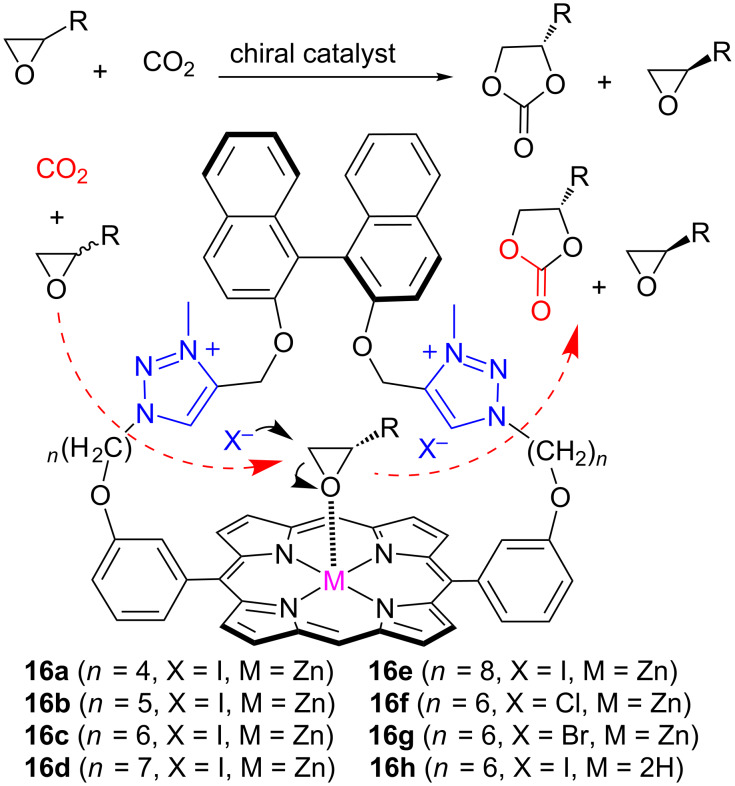
Chemical structure of compound **16** for the kinetic resolution of epoxides.

The condensation of click-reaction-derived triazole containing dialdehyde and binaphthyl with dipyrromethene, followed by zinc metalation and subsequent reaction with MeI resulted in the synthesis of the two-component catalytic system **16** (Figure 13). Here, the cooperative effect of nucleophilic triazolium moieties, their counter ion near the binaphthyl system and the Lewis acidic metal center facilitated the enantioselective synthesis of cyclic carbonates from epoxides. Various catalyst was screened by changing the linker length (*n* = 4 to 8) and nucleophilic counter anion (X = I, Cl, Br), and **16c** was found to be the best catalyst for the reaction with the highest enantioselectivity. The importance of the bifunctional system for the catalytic activity and enantioselectivity was demonstrated by performing a series of reactions with lower activity and selectivity, utilizing free-base porphyrin **16h** with triazolium moieties and the two component catalytic system composed of **16c** before methylation and TBAI as reference catalysts. Optimization studies of the reaction conditions showed that the addition of CHCl_3_ and lowering of the reaction temperature (to 10 °C) considerably increased the enantioselectivity (*s* value of 4.1). An evaluation of the substrate scope showed that various epoxides reacted under the optimized conditions to yield optically active cyclic carbonates and epoxides [63].

### 4. pH sensitive 1,2,3-triazolium macrocycles

Various macrocycles show remarkable response to the pH of the environment. In the following section, we will focus on describing the behavior of three different 1,2,3-triazolium macrocycles including 1,2,3-triazolium catenanes, containing dinaphtho-24-crown-8 (DN24C8) and naphthalenediimide (NDI) moieties at different pH values.

#### 4.1. pH sensors based on 1,2,3-triazolium macrocycles

**Figure 14:**
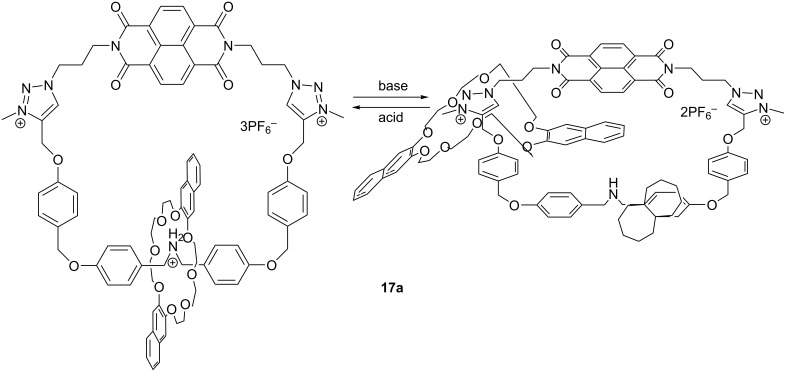
Chemical structure of compound **17a** (bisnaphtho crown ether shown).

Different methods have been recently reported in the literature for the preparation of bistable catenanes and the incorporation of these molecules into an integrated functional supramolecular system. Li et al. have prepared a new catenane **17** (Figure 14) by utilizing a one-step hydrogen-bond and π-donor/π-acceptor template-directed self-assembly procedure via an intramolecular “CuAAC click” reaction in the presence of electron-rich bisnaphtho- or bisbenzo-24-crown-8 ethers. Afterwards, the triazole groups in the catenanes were methylated. The resulting triazolium macrocycle containing catenanes **17a** (DN24C8) and **17b** (DB24C8) are capable of a reversible pH-controlled movement of the crown ether between two stations. UV–vis absorption spectroscopy experiments and ^1^H NMR spectroscopy have shown that the electron-rich crown ether rings and the electron-deficient *N-*methyl-1,2,3-triazolium can interact with each other strongly [64].

Upon addition of diisopropylethylamine (DIEA) to the solution of [2]catenane **17a**, the color of the solution changed from colorless to slightly yellow due to the strong charge transfer between the naphthalene ring of dinaphtho-24-crown-8 (DN24C8) and the naphthalenediimide (NDI) unit, indicating that the DN24C8 unit moved toward the *N*-methyl-1,2,3-triazolium station and was now close to the NDI moiety. This pH-controlled switching process is reversible (e.g., after addition of trifluoroacetic acid the original state is regained). On the other hand, there were no considerable changes in the ^1^H NMR spectra or color changes, upon addition of DIEA to the corresponding triazole analogue (precursor to **17a**) because of only weak interaction between the crown ether and the triazole unit. This clearly proves the importance of the triazolium moiety for the switching process. Color changes with catenane **17b** upon addition of base were less noticeable because of a weaker CT band in the case of a dibenzo-fused crown ether.

Thus, the authors have proven that the movement of macrocyclic rings within catenanes can be controlled by switching the pH, and the bisnaphtho crown ethers can act either as efficient hydrogen-bond acceptors for the secondary dialkylammonium (in acid circumstances) or as binding units for NDI by the π-donor/π-acceptor interaction, which is signalled by a color change.

#### 4.2. pH-sensitive double-lasso molecular machine

**Figure 15:**
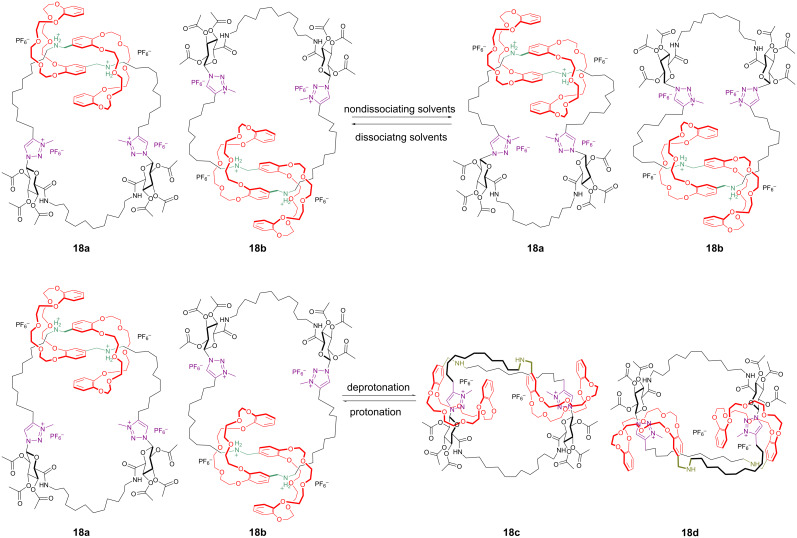
Jump rope in molecular double-lasso compounds **18**.

The synthesis of various rotaxane molecular machines and molecular muscles has recently been reported. Adaptable interlocked double-lasso structures containing 1,2,3-triazolium moieties could be of interest as novel molecular drug carriers capable to release their cargo at a specific pH [65]. In 2012, Coutrot and co-workers have synthesized a novel double-lasso molecular machine **18** (Figure 15) containing two dibenzo-24-crown-8 (DB24C8) surrounding threads, each containing two alternative sites of interactions (also called ‘‘molecular stations’’) for the DB24C8 and they called this a rota-macrocycle in which the rotation of a molecular jump rope can be operated by controlling the translation of the DB24C8 moieties from an ammonium to a triazolium site [66]. In acidic medium, the internal cavity size of the double-lasso structure is large enough to cause an easy movement in dissociating solvents around the [c2] daisy chain (“jump rope” moving from **18a** to **18b**). The nonmethylated precursors of **18** have the same fast kinetic exchange. In nondissociating solvents in acid conditions there is a smaller repulsion between the 1,2,3-triazolium moieties and as a result the U-shape is more tightened than the one in more dissociating solvents such as CD_3_CN or DMSO-*d*_6_, which decreases the kinetic rate of the equilibrium between **18a** and **18b**, making both rotamers observable in the ^1^H NMR spectra.

Conversely, after deprotonation of the ammonium moieties the macrocycle **18a** is forced to adopt a helix-type contracted conformation (two slowly interconverting rotamers **18c** and **18d**) with a reduced cavity. These conformational changes are reversible in solution with switching pH.

#### 4.3. A multivalent catenane and its motion in diverse pH media

**Figure 16:**
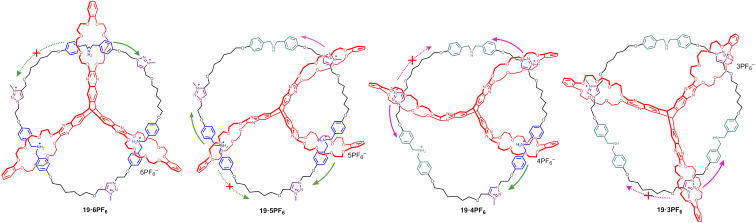
Chemical structure of compound **19** and acid–base triggered motions.

Cell signaling, energy transduction, and cargo delivery are some of functions of very complicatedly assembled biomolecular machines. Multivalent interactions, compared to monovalent receptor–ligand interactions offer the advantage of multiple and sequential binding within the host–guest systems. These noncovalent interactions which can be controlled in a chemical or physical manner depend on the binding sites in the multivalent assembly [67]. Switchable mechanically interlocked molecules (MIM) that rely on multivalency have been studied rarely, thus Chen et al. have reported the design and highly efficacious synthesis of a novel two-component, triply interlocked [2](3)catenane C–H_3_·6PF_6_ **19** (Figure 16) by use of a pyrazine-extended triptycene-derived tris(crown ether) host (eTC) via a dynamic covalent chemistry approach involving triple threading and a Grubbs-catalyzed ring-closing metathesis [68].

^1^H NMR titration experiments have been used for the identification of co-conformations of the four related stable states of **19**. The authors have also determined the dissociation constant p*K*_a_ of this acid−base switchable MIM system by an indicator method which could make a quantitative and precise estimation of its dynamics with environmental acidity change.

By combining NMR spectroscopic data and quantum chemistry calculations, a motion mechanism has been proposed for the MIM macrocycle **19** involving a stepwise co-conformational progression. After the deprotonation of dibenzylammonium (DBA) sites, the rings prefer to move toward the adjacent *N*-methyl-1,2,3-triazolium (MTA) sites to the thermodynamically stable co-conformations rather than migrating to the one which is located at a longer distance, either in a clockwise or counterclockwise sequence. As a single dibenzo-24-crown-8 (DB24C8) ring is not capable to show discriminating recognition for the two MTA sites, the selectivity would be strange indicating that a substantial relation exists between structure topology and mechanical properties [69]. It was argued that carrying out unidirectional 360° rotations, or achieving an all-around mechanostereoselectivity [70] control could be realized by introducing another functional spacer in the [2](3)catenane [71]. Considering that the paths of the B24C8 ring to nearby recognition sites are probably kinetically favorable. Thus, this kind of macrocycles can be used as molecular motors [72] with superior structural and mechanical properties and this could lead to an increased understanding of the mechanism of other complicated biological molecular machines.

## Conclusion

Herein, we have illustrated the recent developments for the synthesis, applications and properties of 1,2,3-triazolium macrocycles. So far, mainly CuAAC reactions have been used for the synthesis of the triazole unit in the macrocycle. In this review we have briefly illustrated the way of utilizing different types of noncovalent interactions including hydrogen bonding, halogen and chalcogen bonding for the selective detection of anions by triazolium macrocycles. The selectivity and the ability of detection of anions are highly dependent on the solvent polarity. We have also described the design and application of 1,2,3-triazolium macrocycles in molecular reactors, pH sensors and drug carriers.

There are still lots of opportunities to design new 1,2,3-triazolium macrocycles as chemosensors for the highly selective recognition of ions in water or biological systems. By introducing a variety of chiral building blocks in the 1,2,3-triazolium macrocycles valuable insight may be provided into the enantioselective recognition and transformation of guest molecules. In the past decades, organocatalytic routes have emerged as an effective and ecofriendly approach towards the synthesis of functionalized and fused triazoles [24]. The methods are easily amenable to chiral natural products [73]. Accordingly, there is a significant potential for introducing these new strategies for the design of triazolium macrocycles.

## References

[1] Desiraju G R (2001). Nature.

[2] Ariga K, Kunitake T (2006). Supramolecular Chemistry – Fundamentals and Applications.

[3] Gale P A, Steed J W (2012). Supramolecular Chemistry: From Molecules to Nanomaterials.

[4] Wen J T, Roper J M, Tsutsui H (2018). Ind Eng Chem Res.

[5] Chen Y, Sun S, Lu D, Shi Y, Yao Y (2019). Chin Chem Lett.

[6] Geng W-C, Sun H, Guo D-S (2018). J Inclusion Phenom Macrocyclic Chem.

[7] Chen Z, Wang Q, Wu X, Li Z, Jiang Y-B (2015). Chem Soc Rev.

[8] Xu Z, Kim S K, Yoon J (2010). Chem Soc Rev.

[9] Yoon J, Kim S K, Singh N J, Kim K S (2006). Chem Soc Rev.

[10] Setsune J-i (2017). Chem Rev.

[11] Vargas-Zúñiga G I, Sessler J L (2017). Coord Chem Rev.

[12] Suk J-m, Chae M K, Kim N-K, Kim U-I, Jeong K-S (2008). Pure Appl Chem.

[13] Black D S C, Craig D C, Kumar N (1989). J Chem Soc, Chem Commun.

[14] Aizpurua J M, Fratila R M, Monasterio Z, Pérez-Esnaola N, Andreieff E, Irastorza A, Sagartzazu-Aizpurua M (2014). New J Chem.

[15] Schulze B, Schubert U S (2014). Chem Soc Rev.

[16] Peng R, Xu Y, Cao Q (2018). Chin Chem Lett.

[17] Hua Y, Flood A H (2010). Chem Soc Rev.

[18] Metrangolo P, Meyer F, Pilati T, Resnati G, Terraneo G (2008). Angew Chem, Int Ed.

[19] Huisgen R, Szeimies G, Möbius L (1967). Chem Ber.

[20] Rostovtsev V V, Green L G, Fokin V V, Sharpless K B (2002). Angew Chem, Int Ed.

[21] Tornøe C W, Christensen C, Meldal M (2002). J Org Chem.

[22] Zhang L, Chen X, Xue P, Sun H H Y, Williams I D, Sharpless K B, Fokin V V, Jia G (2005). J Am Chem Soc.

[23] Ding S, Jia G, Sun J (2014). Angew Chem, Int Ed.

[24] John J, Thomas J, Dehaen W (2015). Chem Commun.

[25] Marx V M, Herbert M B, Keitz B K, Grubbs R H (2013). J Am Chem Soc.

[26] Vasdev R A S, Preston D, Crowley J D (2017). Dalton Trans.

[27] Mirjafari A (2018). Chem Commun.

[28] Vivancos Á, Segarra C, Albrecht M (2018). Chem Rev.

[29] Steed J W (2009). Chem Soc Rev.

[30] Caballero A, Zapata F, González L, Molina P, Alkorta I, Elguero J (2014). Chem Commun.

[31] Hudnall T W, Gabbaï F P (2007). J Am Chem Soc.

[32] Brotherhood P R, Davis A P (2010). Chem Soc Rev.

[33] Davis A P (2006). Coord Chem Rev.

[34] Tamminen J, Kolehmainen E (2001). Molecules.

[35] Gautrot J E, Zhu X X (2009). J Mater Chem.

[36] Chhatra R K, Kumar A, Pandey P S (2011). J Org Chem.

[37] Kumar A, Pandey P S (2008). Org Lett.

[38] Hinman A S, Pavelich B J (1989). J Electroanal Chem Interfacial Electrochem.

[39] Gilday L C, White N G, Beer P D (2012). Dalton Trans.

[40] White N G, Beer P D (2012). Beilstein J Org Chem.

[41] Li S, Liu M, Zhang J, Zheng B, Zhang C, Wen X, Li N, Huang F (2008). Org Biomol Chem.

[42] Liu Y, Flood A H, Bonvallet P A, Vignon S A, Northrop B H, Tseng H-R, Jeppesen J O, Huang T J, Brough B, Baller M (2005). J Am Chem Soc.

[43] Dichtel W R, Miljanić O Š, Spruell J M, Heath J R, Stoddart J F (2006). J Am Chem Soc.

[44] Leigh D A, Wong J K Y, Dehez F, Zerbetto F (2003). Nature.

[45] Ikeda T, Stoddart J F (2008). Sci Technol Adv Mater.

[46] Chmielewski M J, Davis J J, Beer P D (2009). Org Biomol Chem.

[47] White N G, Lovett H G, Beer P D (2014). RSC Adv.

[48] Mercurio J M, Caballero A, Cookson J, Beer P D (2015). RSC Adv.

[49] Barendt T A, Ferreira L, Marques I, Félix V, Beer P D (2017). J Am Chem Soc.

[50] Lim J Y C, Marques I, Thompson A L, Christensen K E, Félix V, Beer P D (2017). J Am Chem Soc.

[51] Kubik S (2010). Chem Soc Rev.

[52] White N G, Carvalho S, Félix V, Beer P D (2012). Org Biomol Chem.

[53] Cai J, Hay B P, Young N J, Yang X, Sessler J L (2013). Chem Sci.

[54] Mesquida N, Dinarès I, Ibáñez A, Alcalde E (2013). Org Biomol Chem.

[55] Lee D Y, Singh N, Kim M J, Jang D O (2011). Org Lett.

[56] Jin S, Kato S-i, Nakamura Y (2016). Chem Lett.

[57] Iwasawa N, Takahagi H, Ono K, Fujii K, Uekusa H (2012). Chem Commun.

[58] Diederich F (1991). Cyclophanes; Monographs in Supramolecular Chemistry.

[59] Li Y, Zhao Y, Jiang R, Liu H, Li Y (2014). Inorg Chem Front.

[60] Guha S, Goodson F S, Corson L J, Saha S (2012). J Am Chem Soc.

[61] Raynal M, Ballester P, Vidal-Ferran A, van Leeuwen P W N M (2014). Chem Soc Rev.

[62] Li Y-j, Zhao Y-j, Flood A H, Liu C, Liu H-b, Li Y-l (2011). Chem – Eur J.

[63] Maeda C, Mitsuzane M, Ema T (2019). Org Lett.

[64] Yang W, Li Y, Zhang J, Chen N, Chen S, Liu H, Li Y (2011). J Org Chem.

[65] Coutrot F, Busseron E (2008). Chem – Eur J.

[66] Romuald C, Ardá A, Clavel C, Jiménez-Barbero J, Coutrot F (2012). Chem Sci.

[67] Badjić J D, Nelson A, Cantrill S J, Turnbull W B, Stoddart J F (2005). Acc Chem Res.

[68] Meng Z, Han Y, Wang L-N, Xiang J-F, He S-G, Chen C-F (2015). J Am Chem Soc.

[69] Barnes J C, Frasconi M, Young R M, Khdary N H, Liu W-G, Dyar S M, McGonigal P R, Gibbs-Hall I C, Diercks C S, Sarjeant A A (2014). J Am Chem Soc.

[70] Fahrenbach A C, Bruns C J, Li H, Trabolsi A, Coskun A, Stoddart J F (2014). Acc Chem Res.

[71] Baroncini M, Silvi S, Venturi M, Credi A (2012). Angew Chem, Int Ed.

[72] Hernández J V, Kay E R, Leigh D A (2004). Science.

[73] Thomas J, Jana S, John J, Liekens S, Dehaen W (2016). Chem Commun.

